# *In silico* Determination of Some Conditions Leading to Glycolytic Oscillations and Their Interference With Some Other Processes in *E. coli* Cells

**DOI:** 10.3389/fchem.2020.526679

**Published:** 2020-10-28

**Authors:** Gheorghe Maria

**Affiliations:** ^1^Department of Chemical and Biochemical Engineering, University POLITEHNICA of Bucharest, Bucharest, Romania; ^2^Chemical Sciences Section, Romanian Academy, Bucharest, Romania

**Keywords:** glycolysis, tryptophan synthesis, *Escherichia coli*, oscillation occurrence, kinetic models, tryptophan synthesis oscillations, bioreactor sensitivity

## Abstract

Autonomous oscillations of species levels in the glycolysis express the self-control of this essential cellular pathway belonging to the central carbon metabolism (CCM), and this phenomenon takes place in a large number of bacteria. Oscillations of glycolytic intermediates in living cells occur according to the environmental conditions and to the cell characteristics, especially the adenosine triphosphate (ATP) recovery system. Determining the conditions that lead to the occurrence and maintenance of the glycolytic oscillations can present immediate practical applications. Such a model-based analysis allows *in silico* (model-based) design of genetically modified microorganisms (GMO) with certain characteristics of interest for the biosynthesis industry, medicine, etc. Based on our kinetic model validated in previous works, this paper aims to *in silico* identify operating parameters and cell factors leading to the occurrence of stable glycolytic oscillations in the *Escherichia coli* cells. As long as most of the glycolytic intermediates are involved in various cellular metabolic pathways belonging to the CCM, evaluation of the dynamics and average level of its intermediates is of high importance for further applicative analyses. As an example, by using a lumped kinetic model for tryptophan (TRP) synthesis from literature, and its own kinetic model for the oscillatory glycolysis, this paper highlights the influence of glycolytic oscillations on the oscillatory TRP synthesis through the PEP (phosphoenolpyruvate) glycolytic node shared by the two oscillatory processes. The numerical analysis allows further TRP production maximization in a fed-batch bioreactor (FBR).

## Introduction

“Autonomous oscillations of species levels in the glycolysis express the self-control of this essential cellular pathway belonging to the CCM, and this phenomenon takes place in a large number of bacteria.

The study of glycolytic oscillations might, therefore, prove crucial for the general understanding of the cell metabolism regulation and the connections among different parts of metabolism. The key question in this context is the mechanism of the oscillations but, despite much work over the last 40 years, it remains unsettled (Wierschem and Bertram, 2004; Madsen et al., 2005).

**Figure 1:**
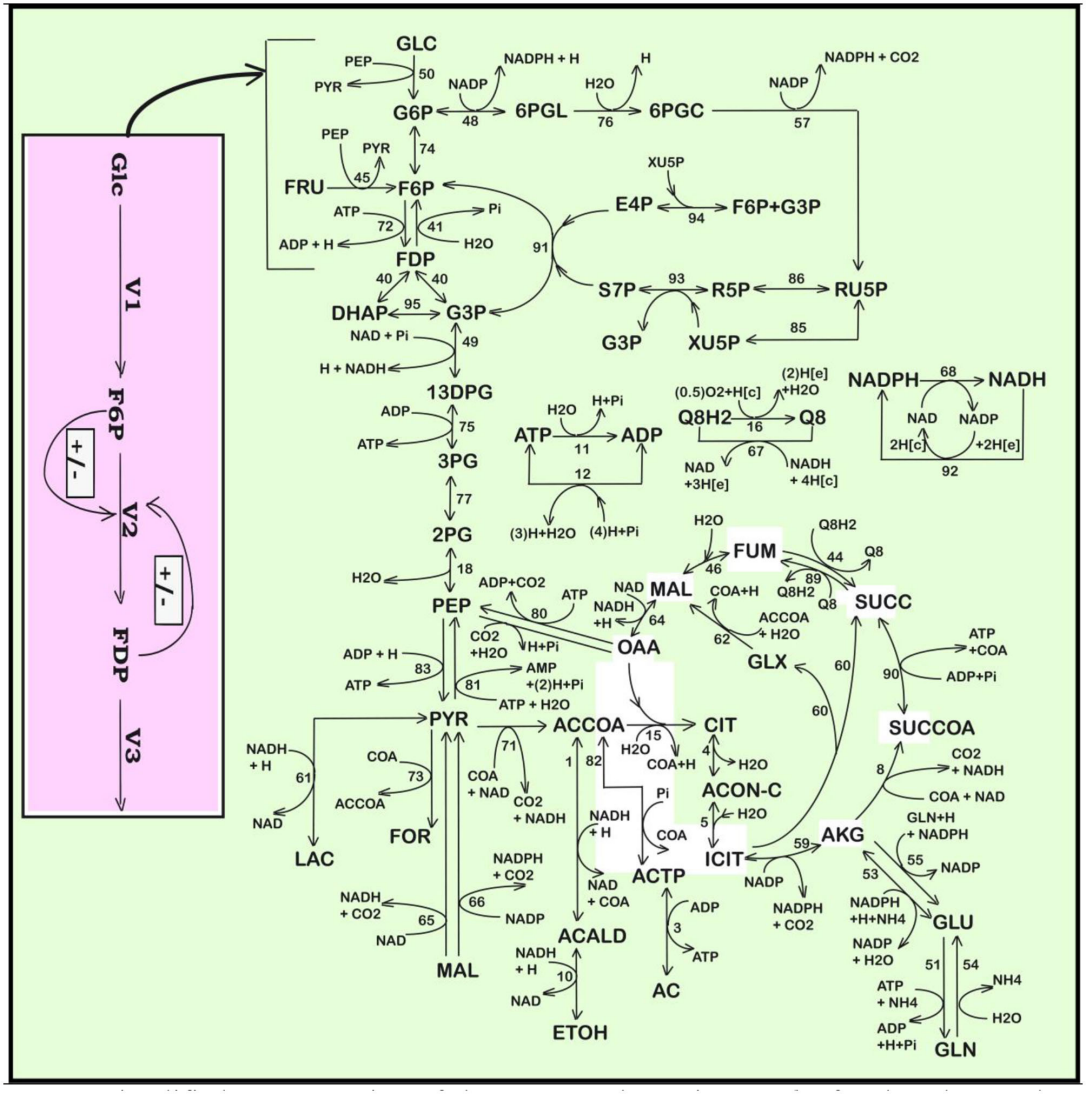
Simplified representation of the CCM pathway in *E. coli* of Edwards and Palsson (2000). Fluxes characterizing the membranar transport [*Metabolite*(e) ↔ *Metabolite*(c)] and the exchange with environment have been omitted from the plot (see Maria et al., 2011 for details, and for explanations regarding the numbered reactions). [e], environment; [c], cytosol. Adapted from Maria et al. (2011, 2018c) courtesy of CABEQ Jl. The considered 72 metabolites, the stoichiometry of the 95 numbered reactions, and the net fluxes for specified conditions are given by Maria et al. (2011). The pink rectangle indicates the chemical node inducing glycolytic oscillations (after Termonia and Ross, 1981a,b; see also Maria et al., 2018c). Notations + and − denote the feedback positive or negative regulatory loops, respectively. Glc, glucose; F6P, fructose-6-phosphate; FDP, fructose-1,6-biphosphate; V1–V3, reaction rates indicated in Figure 2.

**Figure 2:**
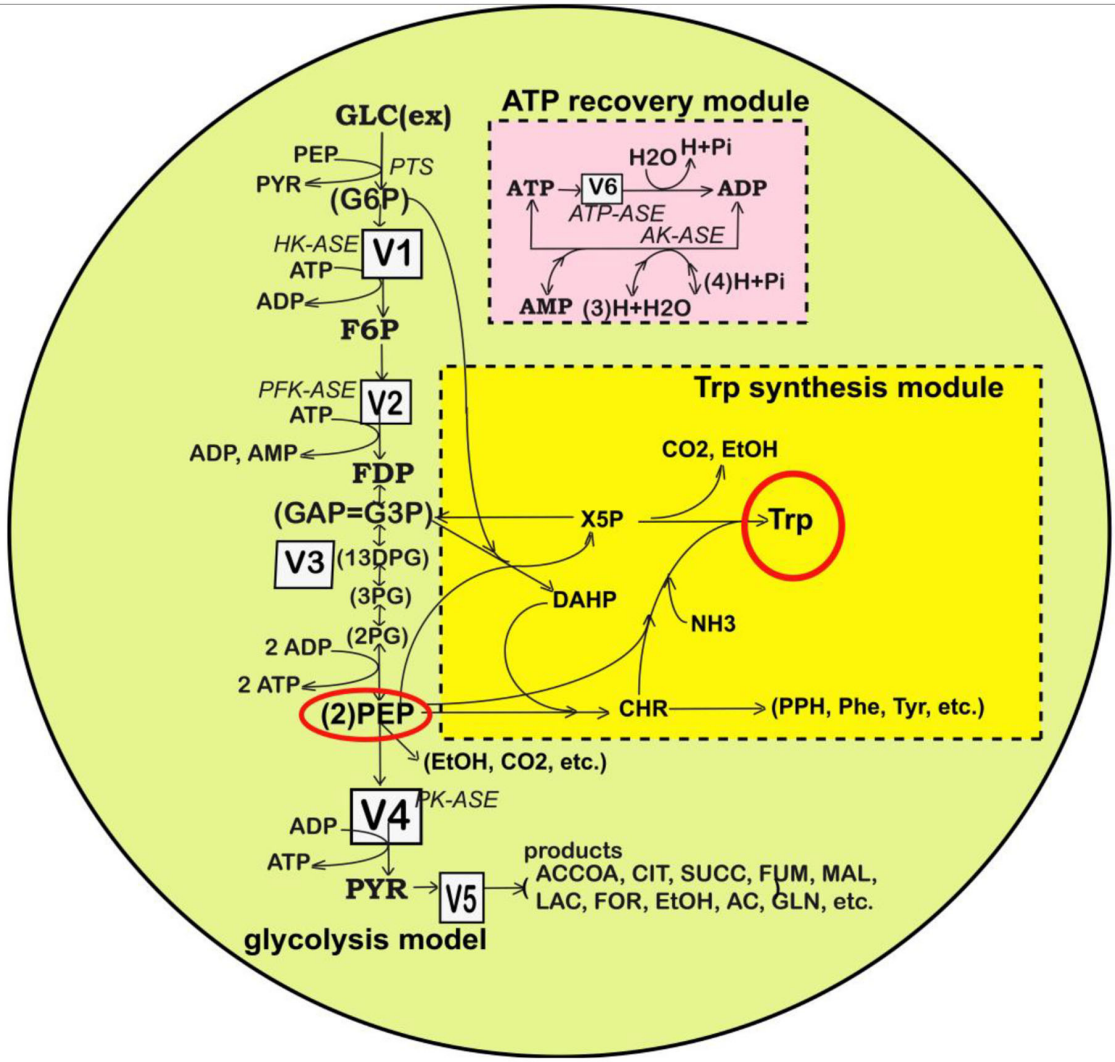
Simplified representation of the structured reaction pathway of glycolysis (Maria, 2014), and of the TRP synthesis (in the yellow area) (Maria et al., 2018a) in *E. coli* used by Maria et al. (2018a,b,c) to derive the process kinetic model and the operating conditions of the fed-batch bioreactor that maximize the TRP synthesis. Connection of the TRP synthesis to glycolysis is realized through the PEP node (Maria et al., 2018c; Mihalachi and Maria, 2019). The modular model structure also includes the adenosine co-metabolites ATP, ADP, and AMP synthesis as part of the ATP recovery system (the pink rectangle). Notations: Species in parenthesis are not explicitly included in the glycolysis model. Italic letters denote the enzymes. Squares include notations of enzymatic reactions V1–V5 included in the glycolysis model. Adapted from Maria (2014; 2018b) courtesy of CABEQ Jl. GLC(ex), glucose in the cell environment; G6P, glucose-6-phosphate; F6P, fructose-6-phosphate; *HK-ASE*, hexokinase; *PFK-ASE*, phosphofructokinase; *ATP-ASE*, ATP monophosphatase; ADP, adenosine diphosphate; ATP, adenosine triphosphate; AMP, adenosine monophosphate; *AK-ASE*, adenylate kinase; Pi, phosphoric acid; FDP, fructose-1,6-biphosphate; G3P,GAP, glyceraldehyde-3-phosphate; 13DPG=PGP, 1,3-diphosphoglycerate; 3PG, 3-phosphoglycerate; 2PG, 2-phosphoglycerate; PEP, phosphoenolpyruvate; PYR, pyruvate; SUCC, succinate; NAD(P)H, nicotinamide adenine dinucleotide (phosphate) reduced; CIT, citrate; ACCOA, acetyl-coenzyme A; LAC, lactate; ETOH, ethanol; AC, acetate.

A model able to simulate the dynamics of the cell CCM must include linked modules relating to (i) the glycolysis (Figures 1, 2); (ii) the phosphotransferase (PTS) system for GLC import into the cell (Figure 1); (iii) the pentose-phosphate pathway (PPP) to generate NADPH and pentoses (5-carbonsugars), as well as ribose 5-phosphate (R5P, a precursor for the synthesis of nucleotides); (iv) the tricarboxylic acid cycle (TCA); (v) the ATP recovery system, and several other pathways” (Palsson, 2005; Kadir et al., 2010; KEGG, 2011; Maria, 2018).

“Modeling bacteria CCM, or parts of CCM, is a subject of very high interest, because the CCM is the essential part of any systematic and structured (model-based) analysis of the cell metabolism with immediate applications, such as biosynthesis optimization, metabolic fluxes evaluation (Stephanopoulos et al., 1998), model-based design of GMO with target characteristics of various applications in the industry, medicine, etc. (Hatzimanikatis et al., 1996; Visser et al., 2004; Styczynski and Stephanopoulos, 2005; Hempel, 2006; Maria and Luta, 2013; Maria, 2018).” Modeling of the CCM kinetics in close connection to the bioreactor environment dynamics falls at the border of several research areas, that is: (bio)chemical physics, computational biochemistry, biochemical engineering, and physical (bio)chemistry.

“To cope with the very high complexity of cell metabolic processes, involving ca. 10^4^ species concentrations, 10^3^ gene expression transcription factors, and ca. 10^5^ enzymatic reactions, adaptable reduced dynamic models, of ‘building-blocks' like modular construction, have been developed over the last decades (reviews of Maria, 2017a,b), with including individual/lumped species and/or reactions.

Modeling the glycolysis dynamics is of particular interest, because most of its intermediates are starting points for the cell production of various metabolites of industrial/medical use (e.g., amino acids, succinate, citrate, etc.; Maria, 2018).

By using two adequate dynamic models validated in previous studies (**Tables 2**, **3**), this paper exemplifies how the model-based analysis can be used (i) to predict some of the internal/environmental conditions inducing glycolytic oscillations in the *Escherichia coli* culture grown in a fed-batch bioreactor (FBR), and (ii) to simulate the influence of the glycolytic oscillations on the TRP oscillatory synthesis by means of the key-species PEP shared by the two oscillatory processes. Industrial applications are immediate seeking for the TRP production optimization. The glycolytic oscillations occurrence will be analyzed vs. external ([GLC]ex in the bulk-phase), and internal factors (that is the ATP recovery rate, dependent on the cell phenotype) (Mathews et al., 1999; Maria, 2014).” The main elements of novelty of this paper consists in the following: (i) Testing and highlighting the influence of the connection of the two metabolic pathways (glycolysis and TRP synthesis), of the external factors ([GLC]ex in the bulk phase, cell dilution rate (μ) adjusted to be equal to the bioreactor dilution rate *D*), and of the cell internal factors (activity of some enzymes in the ATP recovery system) on the two approached oscillatory processes in *E. coli* cells. Several operating policies have been checked in this respect (ii) to eventually suggest how to modulate these most influential factors ([GLC]ex, k6, *D* = μ, and others) before deriving a future optimal operating policy of the FBR (by obtaining, for instance, a timestep-wise GLC feeding policy, not approached here) leading to maximization of the TRP synthesis; (iii) accomplishment of the sensitivity analysis of the studied FBR, which is the preliminary step for any future TRP bioreactor production maximization.

## The Tested FBR Bioreactor

“The *in silico* study of the glycolytic oscillations connected to the TRP synthesis dynamics is performed by considering a FBR with a suspended *E. coli* cell culture, operated with the initial/nominal conditions given in Table 1. It is worth mentioning that the bioreactor includes an excess of sparged air and necessary nutrients for a balanced growth of the cell culture. This FBR was used by Chassagnole et al. (2002) to develop experimental kinetic studies to validate their CCM model (denoted here by CHASSM). The same experiments have also been used by Maria (2014) to validate his reduced kinetic model of glycolysis (denoted here by MGM).”

**Table 1:** The nominal operating conditions of Chassagnole et al. (2002) and Maria et al. (2018a) for the FBR with suspended *E. coli* cell culture used to simulate the glycolytic and TRP synthesis processes.

**The fed-batch (FBR) bioreactor initial conditions**		
**Parameter**	**Nominal value**	**Obs**.
Biomass concentration (*C*_*x*_)	8.7 gDW L^−1^ culture volume	(Chassagnole et al., 2002) assumed to be quasi-constant
Batch time	100 min	
Cell content dilution rate (μ)	10^−4^-10^−2^ min^−1^	To be optimized and adjusted to be identical to *D*
Bioreactor dilution, *D = F_*L*_*/*V_*L*_*	10^−4^-10^−2^ min^−1^ (identical to μ)	To be optimized (nominal 1.667·10^−3^ min^−1^)
Glucose feeding solution concentration $C_{GLC}^{feed}$	100–200, mM	200 mM (this paper) To be optimized
Initial glucose concentration in the bioreactor $C_{GLC}^{ext}\left(t=0\right)$	0.0557–1, mM (Chassagnole et al., 2002)	0.05–1.5 mM (this paper) to be optimized
Biomass density (ρ_*x*_)	565.5 gDW (L cytosol)^−1^	
Initial concentrations for the glycolysis species (in mM)	$c_{GLC}^{ext}\left(t=0\right)$ = 0.0557 *c*_*F*6*P*_(*t* = 0) = 0.600325977 *c*_*FDP*_(*t* = 0) = 0.272961814 *c*_*PEP*_(*t* = 0) = 2.67294507 *c*_*PYR*_(*t* = 0) = 2.67061526 *c*_*ATP*_(*t* = 0) = 4.27 [AMDTP] total = 5.82	Measured by Chassagnole et al. (2002)
Initial concentrations for the TRP synthesis (in μM)	*c*_*OR*_(*t* = 0) = 0.01 *c*_*MRNA*_(*t* = 0) = 0.01 *c*_*E*_ (*t* = 0) = *k*_3_*c*_*MRNA, o*_/μ *c*_*T*_ (*t* = 0) = 0.01	Measured by Bhartiya et al. (2006)

The adopted FBR model of Table 2 “is a classical one (Moser, 1988), developed with the following main hypotheses: (i) the operation is isothermal, iso-pH, and iso-DO (dissolved oxygen); (ii) nutrients are added to the FBR, in recommended quantities, together with an aeration in excess for ensuring an optimal biomass maintenance; (iii) a perfectly mixed liquid phase (with no concentration gradients). The mass balance equations account for the main species in the FBR bulk and of the cellular ones referring to the glycolysis and TRP synthesis dynamics. The resulting ODE model of the FBR (Table 2) also includes the process kinetics (species dynamics). To obtain the species time trajectories with a satisfactory accuracy, by using Table 1 (or other) initial conditions, a low-order stiff integrator (ODE23S) of the Matlab™ software was used” (Maria et al., 2018b,c).

**Table 2:** Species mass balance in the FBR model describing the dynamics of the cellular glycolysis species according to the MGM kinetic model of Maria (2014).

**Mass balance of the main glycolytic species in the living cells of the FBR**	
*GLC* + *PEP* → *F*6*P* + *PYR* *PYR* + *ATP* → *PEP* + *ADP* + *H* *GLC* + *ATP* → *F*6*P* + *ADP* + *H*	$\frac{dc_{GLC}^{ext}}{dt}=D\left(c_{GLC}^{feed}-c_{GLC}^{ext}\right)-\frac{C_{x}}{\rho_{x}}V_{1}$
$V_{1}=r_{PTS}=\frac{\rho_{x}}{C_{x}}\cdot \frac{r_{PTS}^{\max}c_{GLC}^{ext}c_{PEP}/c_{PYR}}{\left(K_{PTS,a1}+K_{PTS,a2}\frac{c_{PEP}}{c_{PYR}}+K_{PTS,a3}c_{GLC}^{ext}+c_{GLC}^{ext}\frac{c_{PEP}}{c_{PYR}}\right)\left(1+\frac{c_{G6P}^{n_{PTS,G6P}}}{K_{PTS,G6P}}\right)}$	
*F*6*P* + *ATP* → *FDP* + *ADP* + *H*	$\frac{dc_{F6P}}{dt}=V_{1}-V_{2}-Dc_{F6P}$
$V_{2}=r_{PFK}=\frac{\left(V_{1}/V_{2m}\right)\;c_{F6P}^{\delta }}{\left(K_{2m}^{\delta }+K_{2m}^{\delta }{\left[\frac{K_{R}^{AMP}}{K_{T}^{ATP}}\right]}^{n}{\left(\frac{c_{ATP}}{c_{AMP}}\right)}^{n}+c_{F6P}^{\delta }\right)}$	
*FDP*+2*ADP*(+2*NAD*+2*P*)⇔2*PEP*+2*ATP*(+2*NADH*+2*H*+2*H*2*O*)	$\frac{dc_{FDP}}{dt}=V_{2}-V_{3}-Dc_{FDP}$
$V_{3}=k_{3}c_{FDP}^{\alpha }-k_{3p}c_{PEP}^{\beta }$	
*PEP* + *ADP* + *H* → *PYR* + *ATP*	$\frac{dc_{PYR}}{dt}=V_{4}-V_{5}-Dc_{PYR}$
$V_{4}=r_{PK}=\frac{\left(V_{1}/V_{4m}\right)c_{PEP}^{\gamma }}{\left(K_{4m}^{\gamma }+K_{4m}^{\gamma }{\left[\frac{K_{R}^{FDP}}{K_{T,PK}^{ATP}}\right]}^{m}{\left(\frac{c_{ATP}}{c_{FDP}}\right)}^{m}+c_{PEP}^{\gamma }\right)}$	
*PYR* → *products*(*ACCOA, CIT, SUCC, LAC, ETOH, AC*, …)	
$V_{5}=\frac{k_{5}c_{PYR}^{n_{consum,PYR}}}{{\text{K}}_{\text{consum,PYR}}+c_{PYR}}$	
*ATP* → *ADP* + *H*	$\frac{dc_{ATP}}{dt}=-V_{1}-V_{2}+2\;V_{3}+V_{4}-V_{6}-Dc_{ATP}$
*V*_6_ = *k*_6_*c*_*ATP*_	
*Obs.:* k6 takes values according to the micro-organism phenotype (characteristics of the gene encoding the enzyme *ATPase* that catalyze this reaction).	
2*ADP*⇔*ATP* + *AMP*	$c_{ATP}c_{AMP}=Kc_{ADP}^{2}$
*Obs.:* (i) Termonia and Ross (1981a,b, 1982) indicated experimental evidence of a very fast reversible reaction catalyzed by *AKase*, the equilibrium being quickly reached.	
(ii) *c*_*AMP*_+*c*_*ADP*_+*c*_*ATP*_ = *c*_*AMDTP*_ = constant (Termonia and Ross, 1981a,b, 1982).	
(iii) *c*_*ADP*_ results from solving the thermodynamic equilibrium relationship $c_{ATP}c_{AMP}=Kc_{ADP}^{2}$, that is: $c_{ADP}^{2}\frac{K}{c_{ATP}}+c_{ADP}-c_{AMDTP}+c_{ATP}=0$	
(iv) Product formation from PYR has been neglected in the model.	
$\frac{dc_{PEP}}{dt}=2\;V_{3}-V_{4}-Dc_{PEP}-y_{trp}\left(2\;V_{3}\right)$	Completion with terms accounting for the PEP consumption in the TRP synthesis: $y_{trp}{=}^{r_{syn,trp}}{/}_{r_{syn,pep}}$ = 1/43.63 (at QSS), from Stephanopoulos and Simpson (1997)

*The glycolysis kinetic model also includes the modification of Maria et al. (2018a) when coupling with the TRP synthesis model. The model parameters are given by Maria (2014; 2018a). Notations are given in the Figure 2 caption*.

## Dynamic Models for the Oscillatory Glycolysis and for the Oscillatory TRP Synthesis in the *E. coli* Cells

### Glycolysis Model

“Glycolysis is a sequence of enzymatic reactions (Figures 1, 2) that converts glucose (GLC) into pyruvate (PYR). The free energy released by the subsequent TCA originating from PYR is used to form the high-energy molecules ATP, and NADH that support the glycolysis and the other enzymatic reactions into the cell (Mathews et al., 1999). Consequently, an adequate modeling/simulation of the glycolysis kinetics is of high importance because its intermediates are entry/exit points to/from glycolysis. For instance, most of the monosaccharides, such as fructose or galactose, can be converted to one of these intermediates. In turn, glycolytic intermediates are directly used in subsequent metabolic pathways. For example, DHAP (an intermediate in the F6P conversion to G3P in Figure 1) is a source of the glycerol that combines with fatty acids to form fat. In addition, NADPH is also formed by the PPP, which converts GLC into R5P, which is used in the synthesis of nucleotides and nucleic acids. PEP is, as well, the starting point for the synthesis of essential amino acids (AA) such as TRP, cysteine, arginine, serine, etc. (Calhoun and Swartz, 2006; Maria et al., 2018a).”

“Due to the huge importance of the glycolysis in simulating the CCM dynamics, intense efforts have been invested both in the experimental study and in modeling of its dynamics in various bacteria (Reeves and Sols, 1973; Bennett et al., 2009; Flamholz et al., 2013; Alberton et al., 2015).”

“However, modeling in detail the glycolysis kinetics and its regulation is a difficult task due to its high complexity. Despite these difficulties, a large number of extended/lumped kinetic models have been proposed (some of them being mentioned in Outline 1) of a complexity varying in the range of 18–30 key species and 48–52 key reactions, with a total of 24–300 or more rate constants. Most of these models are however too complex to be easy to use and identified. Besides, their adequacy is not always satisfactory. Thus, with few exceptions, most of the mentioned models cannot satisfactorily simulate the glycolytic oscillations on a deterministic basis” (Maria, 2014; Mihalachi and Maria, 2019).

**TABLE Outline 1:** Some dynamic models of glycolysis from the literature.

**References**	**Oscill.?**	**Species**	**Reaction**	**Param**.
		**no**.	**no**.	**no**.
Sel'kov (1968)		5	5	?
Termonia and Ross (1981a,b); Termonia and Ross (1982)		9	7	19
Maria (2014) (MGM)		9	6	19
Hatzimanikatis and Bailey (1997)	N	6	9	?
Bier et al. (1996)		7	11	17
Buchholz et al. (2002)	N	3	5	24
Chassagnole et al. (2002) (CHASSM)	N	18	48	127
Westermark and Lansner (2003)		6	6	>30
Degenring et al. (2004)	N	10	22	123
Costa et al. (2008)	N	25	30	116
Costa et al. (2010)	N	18	30	110–116
Kadir et al. (2010)		24	30	>>300
Peskov et al. (2012)	N	48	75+8	>200 (?)

*Some of them include additional modules from the CCM. (N) Indicates the kinetic models not being able to simulate the glycolysis oscillation occurrence (Maria et al., 2011)*.

Starting from the reaction pathway of Figure 1, from the CHASSM and other kinetic models (Outline 1), and by applying advanced lumping algorithms belonging to the physical chemistry (based on species lumping rules with preserving the chemical reaction invariants) (see details of Maria, 2004, 2005), Maria (2014) obtained a reduced kinetic model (that is the MGM) of the glycolysis. The MGM (adopted here) “is accounting for 9 key species, 7 lumped reactions, and includes 17 rate constants (Table 2). Its parameters have been estimated by using the experimental kinetic data of Chassagnole et al. (2002) and Visser et al. (2004).”

The MGM model proved that “it can satisfactorily simulate the dynamics of the glycolytic species concentrations (steady-state QSS, oscillatory, or transient) according to various internal/external regimes, related to (i) the GLC concentration level/dynamics in the bioreactor, (ii) the cell total energy resources in A(MDT)P, and (iii) the cell phenotype responsible for activity of the enzymes involved in the ATP utilization/recovery system. The MGM has been inserted in the bioreactor model template (Table 2) when simulating the dynamics of the [GLC] in the liquid phase simultaneously with that of the cell metabolites. A direct connection between the macro-scale (bioreactor bulk-phase) and the nano-scale (cellular) process variables is thus realized.”

According to Franck (1980), “oscillations in chemical systems represent periodic transitions in time of species concentrations. Thus, spontaneous occurrence of self-sustained oscillations in chemical systems is due the coupled actions of at least two simultaneous processes. Oscillations sourced in a so-called *oscillation node* (that is, a chemical species or a reaction), on which concomitant rapid positive (perturbing) and slow negative (recovering) regulatory loops act (see the discussion of Maria et al., 2018c on the glycolytic oscillation occurrence). Because the coupling action between the simultaneous processes is mutual, the total coupling effect actually forms closed feedback loops for each kinetic variable involved. There exists a well-established set of essential thermodynamic and kinetics prerequisites for the occurrence of spontaneous oscillations, as well as their consequences, extensively discussed by Franck (1980) and Maria et al. (2018c).”

“In the glycolysis case, oscillations is due to the antagonistic action of two processes on regulating the V2 reaction rate (i.e., the oscillation node; Termonia and Ross, 1981a,b, 1982; Maria et al., 2018c). The V2 reaction converts F6P in FDP (see the pink rectangle of Figure 1, including a lumped representation of the glycolytic oscillations node). Glycolytic oscillations properties (period, amplitude) are determined by both external and internal (phenotype) factors. According to Maria (2014) and Maria et al. (2018a,b,c), the glycolysis dynamics [quasi-steady state (QSS) or oscillatory] depends on several factors:”

i) The glucose level in the liquid-phase {denoted by [GLC]ex}, which varies according to the FBR operating conditions;
ii) “The efficiency and the dynamics of the whole ATP recovery system. Among the involved parameters, an essential one is the k6 rate constant (related to the *ATP-ase* characteristics in Figure 2). The involved enzyme characteristics are determined by the cell phenotype (genom) controlling the total energy resources. To not complicate our simulations, the [AMDTP] level was kept unchanged in the present analysis at the value given in Table 1” (Maria, 2004).
iii) “As an important remark, the glycolysis is a systemic process, with a complex regulatory structure. Consequently, oscillations are also related to the rate constants of all the involved reactions, and their appropriate ratios (depending on the enzymes' activity of each microorganism)” (Maria et al., 2018c).

### TRP Synthesis Model

“TRP is an aromatic non-polar α-amino-acid essential in humans that is used in the cell biosynthesis of proteins, being also a precursor to the neuro-transmitter serotonin, of the melatonin hormone, and of vitamin PP. Therefore, maximizing its production via model-based analyses is of particular industrial interest” (Slominski et al., 2002).

“The TRP operon of *E. coli* is one of the most extensively studied molecular regulatory systems (Hernandez-Valdez et al., 2010). The TRP synthesis is known as being an oscillatory process. However, due to the process high complexity, only reduced dynamic models involving lumped reactions/species are used, the regulatory performance being included in adjustable model terms and rate constants. For this reason, in the present analysis, the *in silico* simulation of the TRP synthesis was performed by using the lumped kinetic model of Maria et al. (2018a,b).

This kinetic model is based on the simplified TRP synthesis scheme displayed in Figure 2, derived from various studies reviewed by Maria et al. (2018a). The adopted model for the TRP synthesis, presented in Table 3, is a modification of the Bhartiya et al. (2006) model in order to better fit the experimental kinetic curves of the key species {OR, mRNA, T, E}. Besides, the model was explicitly connected to the glycolysis (as displayed in Figure 2), by including in the TRP mass-balance [i.e., (dc_T_/dt) term in Table 3] a term accounting for the PEP precursor, while the PEP consumption term is included in the PEP balance of the MGM model (Table 2). Other dynamic models for the TRP synthesis module are reviewed by Maria et al. (2018a,b).”

**Table 3:** The TRP synthesis kinetic model of Maria et al. (2018a) modified to be coupled with the glycolysis model.

**Mass balance of the main species involved in the TRP synthesis in the living cells of FBR**	
$\frac{dc_{OR}}{dt}=k_{1}c_{OT}C_{1}\left(T\right)-k_{d1}\;c_{OR}-Dc_{OR}$ $\frac{dc_{MRNA}}{dt}=k_{2}c_{OR}C_{2}\left(T\right)-k_{d2}\;c_{MRNA}-Dc_{MRNA}$ $\frac{dc_{E}}{dt}=k_{3}\;c_{MRNA}-Dc_{E}$ $\frac{dc_{T}}{dt}=k_{4}c_{PEP}C_{3}\left(T\right)\;c_{E}-\frac{g▪T}{T+K_{g}}-Dc_{T}$	$C_{1}\left(T\right)=\frac{K_{i,1}^{n_{H}}}{K_{i,1}^{n_{H}}+T^{n_{H}}}$ $C_{2}\left(T\right)=\frac{K_{i,2}^{1.72}}{K_{i,2}^{1.72}+T^{1.72}}$ $C_{3}\left(T\right)=\frac{K_{i,3}^{1.2}}{K_{i,3}^{1.2}+T^{1.2}}$
Obs. The nitrogen source in the TRP synthesis is considered in excess and included in the *k*_4_ constant. To be connected to the glycolysis kinetic model, the PEP concentration kinetic trajectory generated by the glycolysis model was explicitly included in the TRP synthesis rate.	

*Model parameters are given by Maria et al. (2018a). Notations: TRP, tryptophan; OR, the complex between O and R (aporepressor of the TRP gene); OT, total TRP operon; MRNA, tryptophan mRNA during its encoding gene dynamic transcription and translation; E, enzyme anthranilate synthase; T, TRP; QSS, quasi-steady state*.

## Results and Discussion

### Glycolytic Oscillations

**Figure 3:**
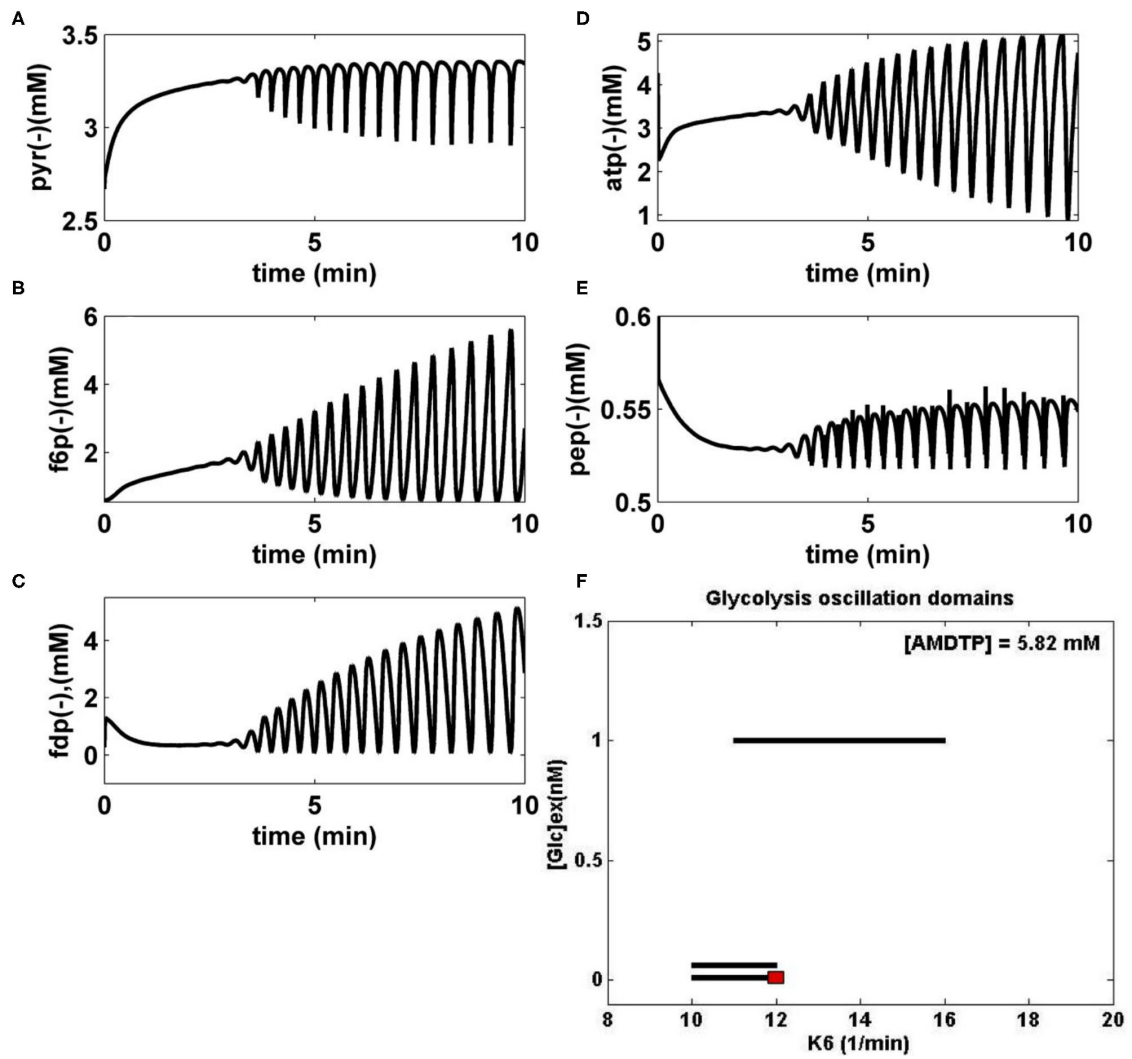
**(A–E)** Simulated glycolytic stationary oscillations of the main glycolytic metabolites (PYR, F6P, FDP, ATP, and PEP) in *E. coli* for the bioreactor nominal operating conditions of Table 1 ([AMDTP] = 5.82 mmol/L, D = 0.001667·1/min), [GLC]ex = 0.0557 mM (at *t* = 0), and k6 = 12 1/min. The simulated FBR running time is 10 min. **(F)** Glycolytic stationary oscillation domains (thick lines) in *E. coli* plotted in the plane [Glc]ext (at *t* = 0), and k6, for the bioreactor nominal operating conditions of Table 1. The red point corresponds to the cell species dynamics plotted in **(A–E)**. [Glc]ex, glucose concentration in the cell environment (bulk phase). Panel **(F)** was adapted from Maria et al. (2018c) courtesy of CABEQ Jl.

Repeated simulations of the bioreactor dynamics using the FBR/MGM kinetic models, with the initial conditions of Table 1 and the parametric ranges of [GLC]ex ∈ [0.01–1.5] mM (at *t* = 0), and k6 ∈ [10–^5^-20] min^−1^ lead to the following results (Maria et al., 2018a,b,c):

i) “Several glycolytic stationary oscillations domains exist in the *E. coli* cells, as indicated by the thick lines of Figure 3F plotted in the {[GLC]ex vs. k6} coordinates.
ii) As displayed in Figure 3F, glycolytic stationary oscillations occur for a slow GLC import not only due to a low [GLC]ex level in the environment but also due to small k6 constant values (that correspond to a low recovery rate of the ATP). Conversely, higher concentrations of GLC in the bioreactor will trigger higher GLC import rates. In this case, glycolytic oscillations are also possible if the k6 constant reported large values (for a certain K constant controlling the AMDTP pathway/equilibrium given in the Table 2). However, the ATP recovery rate is limited by the AMDTP resources and by the interconversion balance of the AMDTP system (Figures 1, 2 and Table 2). As reported by Maria et al. (2018a,b,c), in the cells with too small, or too large k6 values, the glycolysis often reaches its (non-oscillatory) steady state.”
iii) The “glycolytic oscillation domains plotted in Figure 3F are very narrow. Such a result reflects their high sensitivity vs. lots of external and internal factors. Besides, oscillations present a poor stability vs. internal/external factors, as proved by the plotted limit cycles (omitted here; see Maria et al., 2018a,b,c). Experiments in the literature have found that this stability is dependent on the metabolism characteristics of every microorganism. For instance, by contrast, the glycolytic oscillations in yeast have been proved to be very robust even in the presence of environmental noise, with oscillations being a side effect of the trade-offs between robustness and regulatory efficiency of the feedback control of the autocatalytic reaction network (Chandra et al., 2011; Gehrmann et al., 2011).”
iv) The numerical analysis results also indicated that “larger values of k6 lead to a slight decrease in the oscillation period and, eventually, the oscillation disappearance. This is due to the quick consumption of GLC by the cells following a more rapid ATP recovery system (Maria et al., 2018c).”
v) FBR dynamic simulations “have identified glycolytic oscillations with a period of 0.4–1 min, depending on the k6 value and on the [Glc]ex level. For comparison, various experiments in the literature have reported periods in a large range, that is: 0.2 min (Madsen et al., 2005), 2–100 s (Westermark and Lansner, 2003), 15 s (Silva and Yunes, 2006), 1–20 min (Bier et al., 1996), up to 3 h (Rapp, 1979), or 0.2 min to h (Diaz Ricci, 2000).”
vi) The simulated glycolytic oscillations of Figure 3 (FDP and F6P species) are similar to the experimentally recorded dynamics by Schaefer et al. (1999) and Chiarugi et al. (2006) and also similar to the dynamic simulations of Sel'kov (1968), Bier et al. (1996), Elias (2010), and de la Fuente (2010). In fact, Figures 3A–E display an incipient phase of the oscillation occurrence, when the species oscillation amplitude grows. However, over a longer time domain (not shown here), the oscillations stabilize and become stationary.
vii) “The simulated [GLC]ex dynamics in the FBR proved that, for a relatively high [GLC] = 200 mM in the feed, and for all the abovementioned ranges of internal/external operating conditions, the bioreactor evolution is always toward a steady state (QSS), with a faster or slower rate depending on the initial [GLC] in the bioreactor, irrespectively to the cell metabolism (stationary/homeostatic, or unbalanced) (Maria et al., 2018a,b,c).
viii) The factors influencing the glycolysis dynamics mentioned at the end of section Glycolysis Model are confirmed to have a major influence on the glycolysis dynamics as proved by the present analysis.”

### TRP Synthesis Oscillations

“Under certain conditions, the TRP synthesis presents oscillations (Bhartiya et al., 2006). Being strongly connected with the glycolysis (via PEP species), it is important to also study the influence of the glycolytic oscillations on the TRP synthesis dynamics. Such an analysis turns out to be of high practical interest in order to adapt the bioreactor operation to maximize the TRP production and, eventually, for the *in silico* design GMO with such an objective (by the modification of the cell metabolic fluxes) (Maria et al., 2018a,b).

In particular, the glycolysis intermediate PEP is the starting point for the synthesis of essential amino acids, including TRP (Stephanopoulos and Simpson, 1997; Lodish et al., 2000; Calhoun and Swartz, 2006).”

Having PEP as one of the precursors, maximization of TRP synthesis clearly depends on the glycolysis intensity (average levels of glycolytic species) and dynamics (QSS or oscillatory). “On the other hand, as previously discussed, glycolysis is controlled by cell internal and external factors, which indirectly will also influence the TRP synthesis and oscillations, as follows:

(A) The GLC import rate (flux 50 in Figure 1) triggered by the external [GLC]ex and by the PEP and PYR levels into the cell;
(B) The limited ATP energy resources and a slow recovery system can slow down the GLC import, the glycolysis and, implicitly, the all-metabolic syntheses, including the TRP production;
(C) The bacteria genome (cell phenotype) plays an essential role, because it determines the characteristics of the *ATP-ase* and *AK-ase* enzymes responsible for the ATP-to-ADP conversion and for the ATP recovery rate during the glycolytic reactions (accounted by means of the K and k6 rate constants in the MGM kinetic model of Table 2). In fact, the A(MDT)P interconversion system affects most of the metabolic reactions.”
(D) “Being a systemic process, inherently the glycolytic oscillations are also related to the rate constants of all the glycolysis reactions. As an example, Silva and Yunes (2006) found that oscillations are only possible if the [GLC]ex and the maximum reaction rates controlled by the *PFKase* and *GKase* are within specific intervals. The *GKase* is one of the enzymes controlling the V1 lumped reaction related to the PTS import system (GLC to G6P and then to F6P in Figure 2 and Table 2). The *PFKase* controls the V2 reaction (of Figure 2) responsible for the FDP synthesis.”
(E) The results of section Glycolytic Oscillations demonstrated that both external (like [GLC]ex level) and internal/metabolic parameters (like K, k6 constants) governing the glycolytic oscillations will influence the TRP synthesis dynamics and level.

“As has been proven experimentally by Bhartiya et al. (2006), the TRP synthesis is an oscillatory process with a complex engine. Model-based numerical analyses of Maria et al. (2018a,b) highlighted some of the essential factors on which the TRP process dynamics depends.”

As mentioned by Santillan and Mackey (2001); Mackey et al. (2004), and Hernandez-Valdez et al. (2010), “oscillations in the TRP synthesis are produced due to the concomitant activation and high-order repression of the TRP-operon expression, together with a non-linear demand for the end product, making its expression cyclic. Maria et al. (2018a,b) pointed out through model-based simulations that the cell dilution rate (related to the cell cycle), adjusted to be consistent with the liquid residence time in the bioreactor, also exerts a strong influence on the TRP system dynamics.”

**Figure 4:**
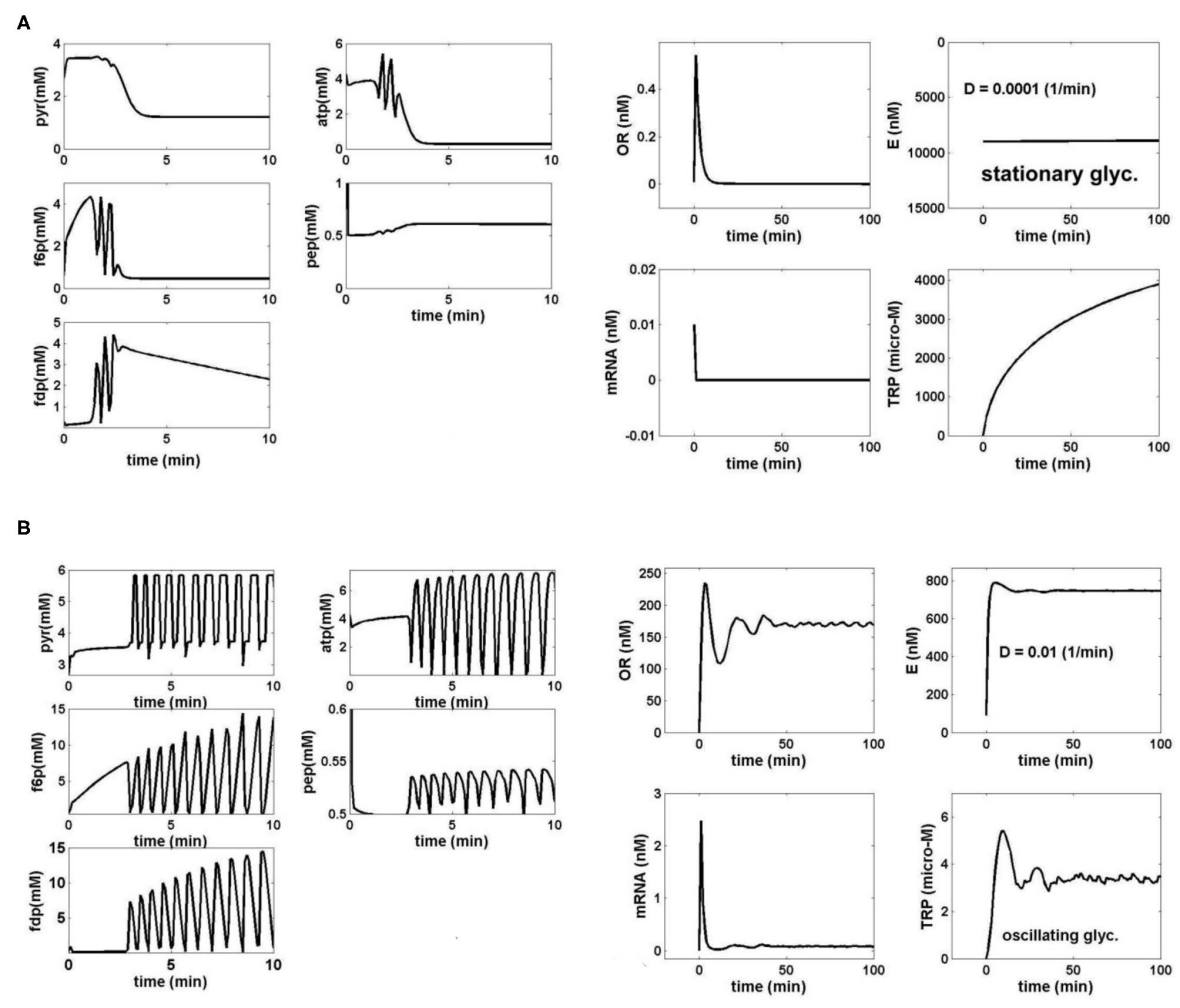
**(A)** Simulated dynamics of the coupled glycolysis and TRP synthesis main species in *E. coli* cells under the bioreactor nominal conditions of Table 1, for a cell phenotype with k6 = 12 1/min. The following operating conditions have been checked: *D* = 0.0001 (1/min) (cell/bioreactor dilutions); [GLC]ex = 1 mM (at *t* = 0), when both glycolysis [left] and TRP synthesis [right] display a stationary behavior (quasi-steady-state, QSS). The realized TRP production over the batch time is 0.39 μM/min. TRP production (μM/min) = {Dilution rate (*F*_*L*_/*V*_*L*_)} × {max [TRP] (*t*)} Species dynamics was generated by using the coupled bioreactor/glycolysis/TRP models of Tables 2, 3. **(B)** Simulated dynamics of the coupled glycolysis and TRP synthesis main species in *E. coli* cells under the bioreactor nominal conditions of Table 1, for a cell phenotype with k6 = 12 1/min. The following operating conditions have been checked: *D* = 0.01 (1/min) (cell/bioreactor dilutions); [GLC]ex = 0.0557 mM (at *t* = 0), when both glycolysis [left] and TRP synthesis [right] display stationary oscillations. The realized TRP production over the batch time is 0.1085 μM/min. TRP production (μM/min) = {Dilution rate (*F*_*L*_/*V*_*L*_)} × {max [TRP] (*t*)} The species dynamics were generated by using the coupled bioreactor/glycolysis/TRP models of Tables 2, 3.

The numerical analysis of the present paper has been performed by using the FBR reactor model, which includes the *coupled* glycolysis MGM model (section Glycolysis Model; Table 2) and the TRP synthesis kinetic model (section TRP Synthesis Model; Table 3). “The sensitivity analysis of the TRP production was performed by considering some of the most influential parameters checked in the range of [GLC]ex ∈ [0.01–1.5] mM (at *t* = 0); k6 ∈ [10^−5^-20] min^−1^; *D* ∈ [10^−4^-10^−2^] min^−1^; and initial [GLC]ex ∈ [0.005–5] mM (at *t* = 0). The simulation results for only two relevant operating conditions have been plotted in Figures 4A,B. This analysis leads to several results and conclusions:

i) The [GLC]ex, the constant k6, and the bioreactor dilution *D* (considered equal to the cell dilution) exert the highest influence not only on the glycolysis dynamics but also on the TRP synthesis dynamics and production (due to its close link to the glycolysis via PEP). Thus, under the initial FBR conditions of Table 1, for a low FBR dilution rate (*D*), and for conditions leading to a QSS glycolysis, the TRP synthesis also displays a stationary evolution (Figure 4A). By contrast, at higher dilutions, and when glycolysis meets the conditions necessary for an oscillatory process (of Figure 3F), the TRP synthesis also presents an oscillatory dynamics (Figure 4B). Consequently, the bioreactor dilution presents a strong influence on the QSS or oscillatory regime of the linked glycolysis and TRP synthesis. The TRP production (see its definition in the caption from Figures 4A,B) is influenced accordingly. A value of k6 = 12 min^−1^ was considered in all the tested cases here.
ii) While glycolysis exerts a strong influence on the TRP synthesis dynamics, as proved by Figures 4A,B, the reverse influence is minor, as proved by disconnected glycolysis simulations (not reproduced here).
iii) For the high feeding rates (Table 1), the initial [GLC] in the FBR bioreactor does not quantitatively influence the TRP bioreactor performances (Maria et al., 2018b).
vi) Simulations of the only TRP synthesis, disconnected from the glycolytic process, but with employing various [PEP] average levels (Mihalachi and Maria, 2019), indicated that PEP average level has a huge influence on the dynamics and concentrations of the TRP synthesis species. In turn, [PEP] is controlled by the glycolysis dynamics, which, in turn, is controlled by the abovementioned external and internal factors.
v) It clearly appears that, beside cell phenotype (governing the TRP operon expression), glycolysis is one of the major factors influencing the TRP production. Thus, by ranging the FBR operating parameters, the TRP production can be maximized (Maria et al., 2018b).
vi) In all the FBR operating cases checked by Maria et al. (2018b) with the initial conditions of Table 1, but in the range of *D* ∈ [10^−4^-0.01] min^−1^, simulations demonstrated that [GLC]ex in the liquid bulk always evolves toward its steady state irrespectively of the stationary or oscillatory dynamics of the cell metabolic processes.”

### Engineering Implications—Sensitivity Analysis

From an engineering point of view, the study suggests how to modulate the most influential factors, that is: [GLC]ex {via [GLC](feed), and [GLC](initial)}, *D* = μ, k6, and others (via cell phenotype), before deriving an optimal FBR operating policy (for instance, a timestep-wise GLC feeding policy) leading to maximization of the TRP synthesis.

**Figure 5:**
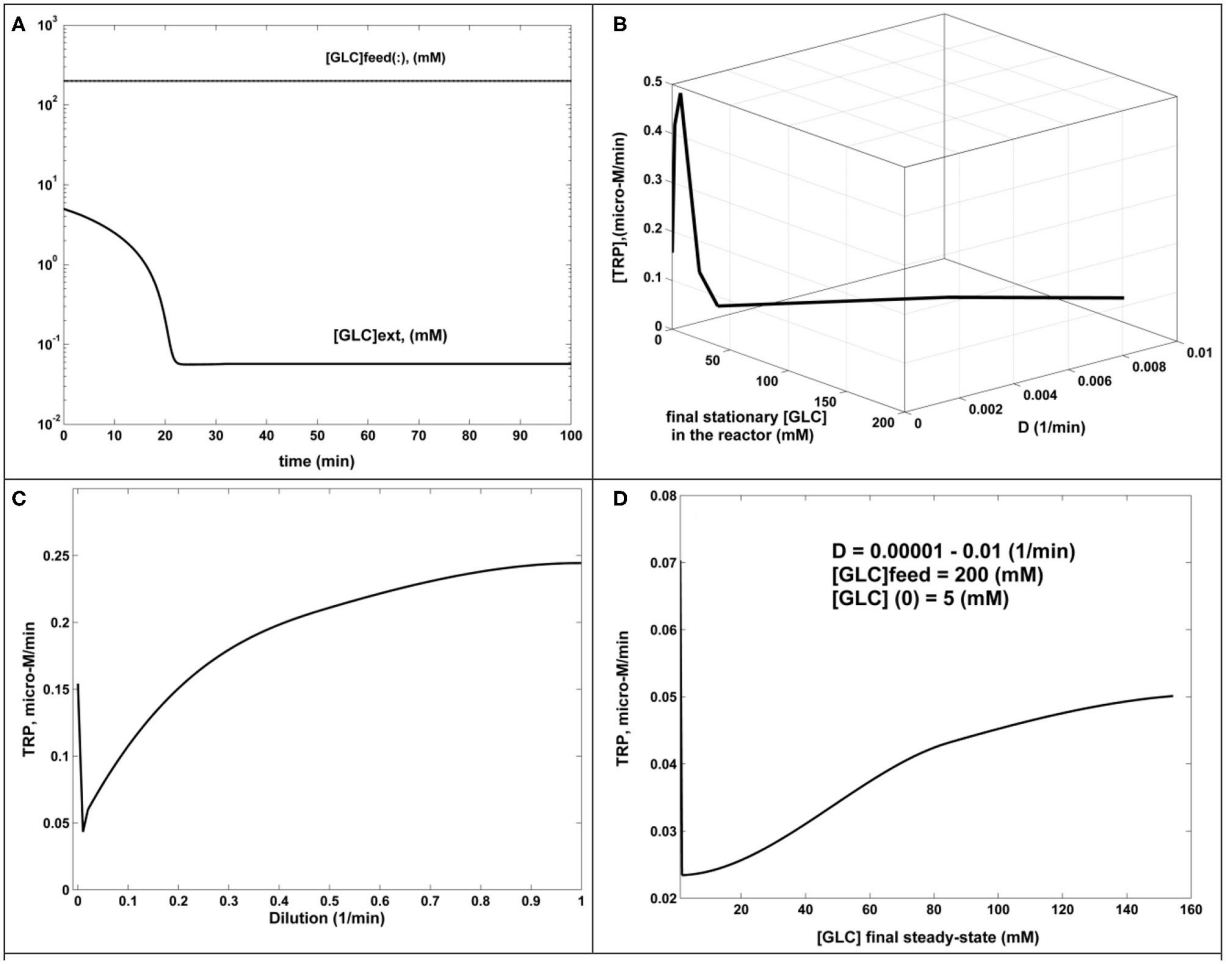
The dynamics and sensitivity analysis of the FBR (simulated results). **(A)** [GLC] dynamics in the FBR for [GLC](feed) = 200 mM; [GLC](initial) = 5 mM; *D* = 0.001 (1/min). The same behavior for *D* = 0.00001–0.001 (1/min). **(B)** TRP production (μM/min) function of dilution and the [GLC](stationary) at the steady state. Fixed parameters of [GLC](feed) = 200 mM; [GLC](initial) = 5 mM. **(C)** TRP production (μM/min) function of reactor dilution. Fixed parameters: of [GLC](feed) = 200 mM; [GLC](initial) = 5 mM. **(D)** TRP production (μM/min) function of [GLC](stationary) at the steady state, generated for dilutions in the range of 0.00001–0.01 (1/min). Fixed parameters of [GLC](feed) = 200 mM; [GLC](initial) = 5 mM.

An exhaustive or an adaptive model-based search can identify the FBR optimal operating policy that corresponds to a maximum of TRP production (an analysis not developed in this work). In the present study, a brief sensitivity analysis based on the above model has been performed, thus preceding the FBR future optimization. Numerical simulations revealed several interesting conclusions:

a.- FBR simulations using various operating parameters proved that FBR efficiency (TRP production) is not influenced by [GLC](initial) < 100 mM in the bioreactor, once [GLC](feed) > 100 mM.
b.- In all tested cases covering the ranges [GLC](feed) of 100–200 mM, [GLC](initial) of 1–50 mM, and *D* = 0.00001–0.01 (1/min), the FBR rapidly evolves toward its steady state, corresponding to a usually small [GLC](stationary) (below 1 mM), which proves the FBR efficiency. An example of GLC dynamics in the bioreactor is displayed in Figure 5A.
c.- The TRP productivity increases with *D*, as plotted in Figure 5C.
d.- The TRP productivity also increases with the [GLC]ex, as plotted in Figure 5D, where the external (bulk) concentration is given by the steady-state level of [GLC](stationary). The combined dependency of TRP productivity on the reactor dilution and the [GLC](stationary) is given in Figure 5B, confirming conclusions (c) and (d) for a wide range of the reactor dilutions.

As proved in this work, the TRP productivity is also strongly dependent on the oscillatory characteristics of the glycolysis, determined by the abovementioned operating parameters, and on the activity of enzymes involved in the ATP recovery system (that is, the rate constants k6, K, and [AMDTP] of Table 2). Thus, from a biological point of view, as mentioned by Silva and Yunes (2006), “glycolytic oscillations are focused on the maintenance of energy levels in the cell (negative regulation of *PFKase* by ATP) and thus the ability to limit the conversion into energy in situations where it is not needed. Therefore, it would be more advantageous to store it or deviate the flux toward other cell cycle activities such as cell division. Consequently, GMO with modified enzyme activity (especially those related to the ATP use/recovery system of Figure 2) will lead to noticeable modifications in the metabolic species dynamics and concentrations.”

## Conclusions

The *in silico* analysis of this paper demonstrates in a meaningful and relevant way the importance of using a detailed enough and adequate structured dynamic model linking the metabolic cellular processes and the bioreactor state variables for engineering purposes. Such a modular hybrid model can link the macro-scale (bioreactor liquid phase) process variables to the nano-scale (cellular) ones.

The structured model not only can be used for future FBR optimization, “but can also be a valuable tool to evaluate the cellular metabolic fluxes (i.e., the homeostatic metabolic reaction rates, not evaluated here), thus opening the possibility to *in silico* re-design the cell metabolism to obtain GMOs with industrial or medical applications (Hatzimanikatis et al., 1996; Stephanopoulos et al., 1998; Visser et al., 2004; Styczynski and Stephanopoulos, 2005; Maria, 2017a,b, 2018).

Thus, the large experimental and computational effort to validate such structured cell models is eventually fully justified through the practical advantages offered by such an engineering analysis.

It is also to be emphasized that such a modular and structured approach of the dynamic cellular models offers the possibility to study the interference of the CCM sub-process (e.g., glycolysis and AA synthesis here), together with the influence of the external conditions. Such a modular simulation platform presents the advantage to be easy to extend by the inclusion of new CCM modules.”

## Data Availability Statement

All datasets generated for this study are included in the article/supplementary material.

## Author Contributions

The author confirms being the sole contributor of this work and has approved it for publication.

## Conflict of Interest

The author declares that the research was conducted in the absence of any commercial or financial relationships that could be construed as a potential conflict of interest.

## Figures and Tables

**Table:** 

*c*_*j*_, *C*_*J*_, **[J]**	species “**j, J**″ concentration	**Indices**	o = initial; syn = synthesis; x = biomass; ≪s≫= stationary (quasi-steady state, QSS)
*D*	bioreactor dilution, *D = F_L_/V_L_*	ρ_*x*_	Biomass density
*F_L_*	liquid feed flow rate in the bioreactor	μ	Cell content dilution rate
*k*_*j*_, *K*_*j*_	rate constants	**Indices**	O = initial; ex, ext = external; syn = synthesis; x = biomass; s = stationary (quasi-steady state, QSS)
*t, t*_*c*_	time, cell cycle		
*V_L_*	liquid volume in the bioreactor	**Super-scripts**	
*y*_*trp*_	stoichiometric coefficient	*n*	reaction order”

**Table:** 

13DPG, PGP	1,3-diphosphoglycerate
3PG	3-phosphoglycerate
2PG	2-phosphoglycerate
AA	Amino-acids
ACCOA	Acetyl-coenzyme A
AC	Acetate
ADP	Adenosine diphosphate
AK-ASE	Adenylate kinase
AMP	Adenosine monophosphate
ATP	Adenosine triphosphate
ATP-ASE	ATP monophosphatase
CCM	Central carbon metabolism
CHASSM	The kinetic model of Chassagnole et al. (2002)
CIT	Citrate
DHAP	Dihydroxyacetone
DO	Dissolved oxygen
DW	Dry mass
E	Enzyme anthranilate synthase in the TRP model
ETOH	Ethanol
FBR	Fed batch
FDP	Fructose-1,6-biphosphate
F6P	Fructose-6-phosphate
G3P,GAP,3PG	Glyceraldehyde-3-phosphate
G6P	Glucose-6-phosphate
GLC	Glucose
GLC(EX)	Glucose in the external environment
GMO	Genetically modified microorganisms
HK-ASE	Hexokinase
LAC	Lactate
MGM	The glycolysis model of Maria (2014)
MRNA	Tryptophan messenger ribonucleic acid during its encoding gene dynamic transcription, and translation
NAD(P)H	Nicotinamide adenine dinucleotide (phosphate) reduced
ODE	Ordinary differential equations
OME	Order of magnitude estimate
OR	The complex between O and R (aporepressor of the TRP gene)
OT	The total TRP operon
P, Pi	Phosphoric acid
PEP	Phosphoenolpyruvate
13DPG=PGP	1,3-diphosphoglycerate;
PFK-ASE	Phosphofructokinase
PK-ASE	Pyruvate kinase
PPP	Pentose-phosphate pathway
PTS	Phosphotransferase, or phosphoenolpyruvate:glucose, or phosphotransferase system
PYR	Pyruvate
QSS	Quasi-steady state
R5P	Ribose 5-phosphate
SBR	Semi-continuous (semi-batch) reactor
SUC, SUCC	Succinate
TCA	Tricarboxylic acid cycle
TF	Gene expression transcription factors
TRP	Tryptophan
Wt.	Weight
